# Sociodemographic and behavioral determinants of *Plasmodium vivax*-specific antibody responses among short-term Myanmar migrants in Thailand

**DOI:** 10.1186/s12936-026-05921-0

**Published:** 2026-04-29

**Authors:** Piyarat Sripoorote, Nichakan Inthitanon, Yupaporn Wattanagoon, Pailene S. Lim, Liwang Cui, Jetsumon Sattabongkot, Daniel M. Parker, Wang Nguitragool, Sadudee Chotirat, Rhea J. Longley, Pyae Linn Aung

**Affiliations:** 1 Mahidol Vivax Research Unit, Faculty of Tropical Medicine, Mahidol University, Bangkok, Thailand; 2 Department of Clinical Tropical Medicine, Faculty of Tropical Medicine, Mahidol University, Bangkok, Thailand; 3 Infection and Global Health Division, WEHI, Parkville, VIC Australia; 4 Department of Medical Biology, University of Melbourne, Parkville, VIC Australia; 5 Division of Infectious Diseases and International Medicine, Department of Internal Medicine, Morsani College of Medicine, University of South Florida, 3720 Spectrum Boulevard, Suite 304, Tampa, FL 33612 USA; 6 Department of Population Health and Disease Prevention, Department of Epidemiology & Biostatistics, University of California, Irvine, CA USA; 7 Department of Molecular Tropical Medicine and Genetics, Faculty of Tropical Medicine, Mahidol University, Bangkok, Thailand

**Keywords:** *Plasmodium vivax*, Seropositivity, protein markers, Risk factors, Thailand, GMS

## Abstract

**Background:**

Migrant populations from malaria-endemic countries pose challenges to elimination efforts in low-transmission settings. This study assessed *Plasmodium vivax*-specific antibody responses and associated sociodemographic and behavioral determinants among short-term Myanmar migrants in Thailand.

**Methods:**

A cross-sectional survey was conducted in March–April 2025 among 300 short-term Myanmar migrants in six malaria high-burden villages of Tha Song Yang District, Tak Province. Sociodemographic and behavioral data were collected via structured questionnaires. Finger-prick dried blood spots were collected to quantify IgG responses to eight *P. vivax* antigens using a multiplexed Luminex assay. Predicted recent exposure was defined using a previously developed and validated random forest classification algorithm, applying a 95% specificity threshold. Logistic regression models were used to identify factors associated with predicted recent exposure, while linear regression models examined associations with a composite antibody score representing overall seroreactivity across multiple antigens.

**Results:**

Among 294 PCR-negative participants, 104 (35.4%) were predicted as recently exposed. In multivariable logistic models, higher odds of predicted recent exposure were observed for age 30–39 years (aOR = 3.11, 95% CI 1.15–8.80), secondary education or above (aOR = 3.28, 95% CI 1.09–10.3), and Karen ethnicity (aOR = 3.68, 95% CI 1.42–10.0). Protective factors included seasonal (aOR = 0.28, 95% CI 0.08–0.95) and temporary work (aOR = 0.26, 95% CI 0.08–0.84), and income > 5,000 THB (aOR = 0.17, 95% CI 0.06–0.45). Exposure was also higher among those living > 15 min from a health facility (aOR = 2.07, 95% CI 1.00–4.43) and with negative malaria attitudes (aOR = 2.02, 95% CI 1.04–4.03). Higher composite antibody levels were observed among males (β = 1.17, 95% CI 0.07–2.28, *p* = 0.038), older participants (β = 0.12/year, 95% CI 0.08–0.17, *p* < 0.001), and agricultural workers (β = 2.58, 95% CI 0.01–5.14, *p* = 0.049). In contrast, higher income (β = − 3.83 per 10,000 THB, 95% CI − 6.28 to − 1.37, *p* = 0.002), longer stay (β =  − 0.02/day, 95% CI − 0.04 to − 0.00, *p* = 0.025), and weekly returns to Myanmar (β = − 2.44, 95% CI − 4.14 to − 0.74, *p* = 0.005) were associated with lower antibody levels.

**Conclusions:**

Among PCR-negative short-term migrants, one-third showed evidence of predicted recent *P. vivax* exposure. Antibody levels varied by demographic, occupational, and mobility-related factors, supporting the use of sero-surveillance to inform border elimination strategies.

**Supplementary Information:**

The online version contains supplementary material available at 10.1186/s12936-026-05921-0.

## Background

Among the six countries in the Greater Mekong Subregion (GMS), both Thailand and Myanmar reported a resurgence of malaria cases from 2021 onward. In Myanmar, malaria cases tripled from approximately 79,000 in 2021 to 228,567 by 2023 [1]. This may represent an underestimate, as surveillance efforts have been drastically disrupted since the coup d'état in 2021. Similarly, Thailand documented over 16,000 cases in 2023, representing a nearly fourfold increase from 2021 [1, 2]. More than half of the reported cases in Thailand were classified as imported, attributable primarily to long- and short-term migrants from Myanmar, potentially driven by ongoing cross-border movements amid political instability [2]. Concurrently, challenges such as the emergence of antimalarial drug resistance, the increasing predominance of *Plasmodium vivax*, prolonged treatment requirements, and limited effectiveness of vector control strategies are impeding progress toward the national malaria elimination goal by 2030 [1, 3–6]. Although *P. falciparum* has historically contributed substantially to malaria burden in the GMS, *P. vivax* has become increasingly important in Thailand and other low-transmission settings approaching elimination [1]. This is partly due to its biological and operational challenges, including relapse from dormant liver-stage hypnozoites, lower-density and asymptomatic infections, and more limited effectiveness of routine surveillance tools for detecting recent exposure. In addition, validated serological markers and a recent-exposure classification algorithm are currently well developed for *P. vivax*, making this species particularly suitable for sero-surveillance applications in mobile populations.

Northwestern Thailand, particularly areas bordering Myanmar, has historically remained endemic for malaria [2]. The presence of competent malaria vectors, including *Anopheles minimus*, *Anopheles dirus*, and *Anopheles maculatus*, facilitates receptivity in the region, and imported infections from Myanmar migrants may therefore perpetuate localized transmission cycles [7–9]. While cross-border movement plays a role in parasite reintroduction, sustained transmission on the Thai side likely reflects a combination of reintroduction and diminished system resilience, potentially due to reduced funding and decreased vigilance following overall declines in malaria burden. According to the International Organization for Migration (IOM), Thailand hosts approximately four to five million migrants, with the majority originating from Myanmar. These individuals are broadly categorized into M1 (long-term migrants, ≥ 6 months) and M2 (short-term migrants, < 6 months) groups [10]. M2 migrants, in particular, experience substantial barriers to accessing malaria services in Thailand, despite the policy of free diagnosis and treatment irrespective of nationality or documentation status. Documented challenges include language barriers, fear of deportation, perceived costs, and low prioritization for malaria relative to other survival needs [11–16]. Even among those who access appropriate care, adherence to the full 14-day primaquine regimen for *P. vivax* is frequently suboptimal, contributing to persistent liver-stage hypnozoites and recurrent infections [17–21]. While individuals with confirmed *P. vivax* infection are typically assumed to harbor dormant liver-stage parasites and are treated with primaquine, no point-of-care diagnostic currently exists to directly detect hypnozoites. The development of such diagnostics could enhance targeted treatment strategies, especially for asymptomatic individuals or those with a prior history of infection who may otherwise go untreated under current protocols. Short-term migrants represent a particularly important population for malaria elimination efforts in Thailand. Unlike long-term migrants or local residents, individuals who have recently crossed the border may not yet be integrated into local health systems, census registries, or routine malaria surveillance activities [16]. As a result, they may be underrepresented in case detection programs and preventive interventions such as insecticide-treated net distribution or community health outreach. Understanding malaria exposure patterns among short-term migrants is therefore critical for identifying potential reservoirs of infection and for informing targeted surveillance and intervention strategies in border regions.

Prolonged residence in endemic areas may increase the likelihood of repeated malaria exposure, potentially contributing to the development of naturally acquired immunity [22–25]. While such immunity does not confer sterilizing protection, it often results in reduced symptom severity and asymptomatic parasitemia. In these cases, individuals may unknowingly harbor parasites and serve as reservoirs for continued transmission, especially in settings with limited access to diagnostic and treatment services [23, 26]. In this context, quantifying *P. vivax*-specific antibody responses offers a valuable surveillance tool to estimate recent exposure and to inform targeted interventions such as serological test-and-treat (sero-TAT) [27]. Numerous *P. vivax* antigens have been identified and validated [28], with several markers reliably classifying recent exposure within the past nine months [29]. Because serological classification algorithms identify exposure within the preceding nine months, while short-term migrants typically reside in Thailand for less than six months, some predicted recent exposure detected by this approach may reflect infections acquired prior to migration from Myanmar. Therefore, serological findings should be interpreted as indicators of recent exposure history rather than definitive evidence of transmission occurring exclusively within Thailand.

Sociodemographic characteristics are known to influence serological responses. For instance, males may exhibit higher seroprevalence due to occupational exposure, and older individuals may accumulate antibody responses over repeated exposures [30–32]. However, no comprehensive assessment of both the magnitude and sociodemographic or behavioral determinants of *P. vivax*-specific antibody responses has been reported among high-risk mobile populations. A PubMed search conducted in November 2025 using combinations of the terms “*Plasmodium vivax*”, “serology”, “antibody”, “migrant”, “mobile population”, and “Thailand” did not identify studies examining sociodemographic or behavioral determinants of *P. vivax*-specific antibody responses among short-term migrant populations in Thailand. Understanding the antibody response profile and its associated risk factors is critical for guiding surveillance and intervention strategies, particularly in border settings where mobile populations sustain residual transmission. Therefore, this study aimed to quantify *P. vivax*-specific antibody responses and identify sociodemographic and behavioral factors associated with recent exposure among short-term Myanmar migrants in northwestern Thailand.

## Methods

### Study design

This cross-sectional study was conducted from March to April 2025 to assess *P. vivax* serological markers of predicted recent exposure (< 9 months) among short-term Myanmar migrants residing in Thailand. Data collection took place at the community level through structured household visits.

### Study sites

**Fig. 1:**
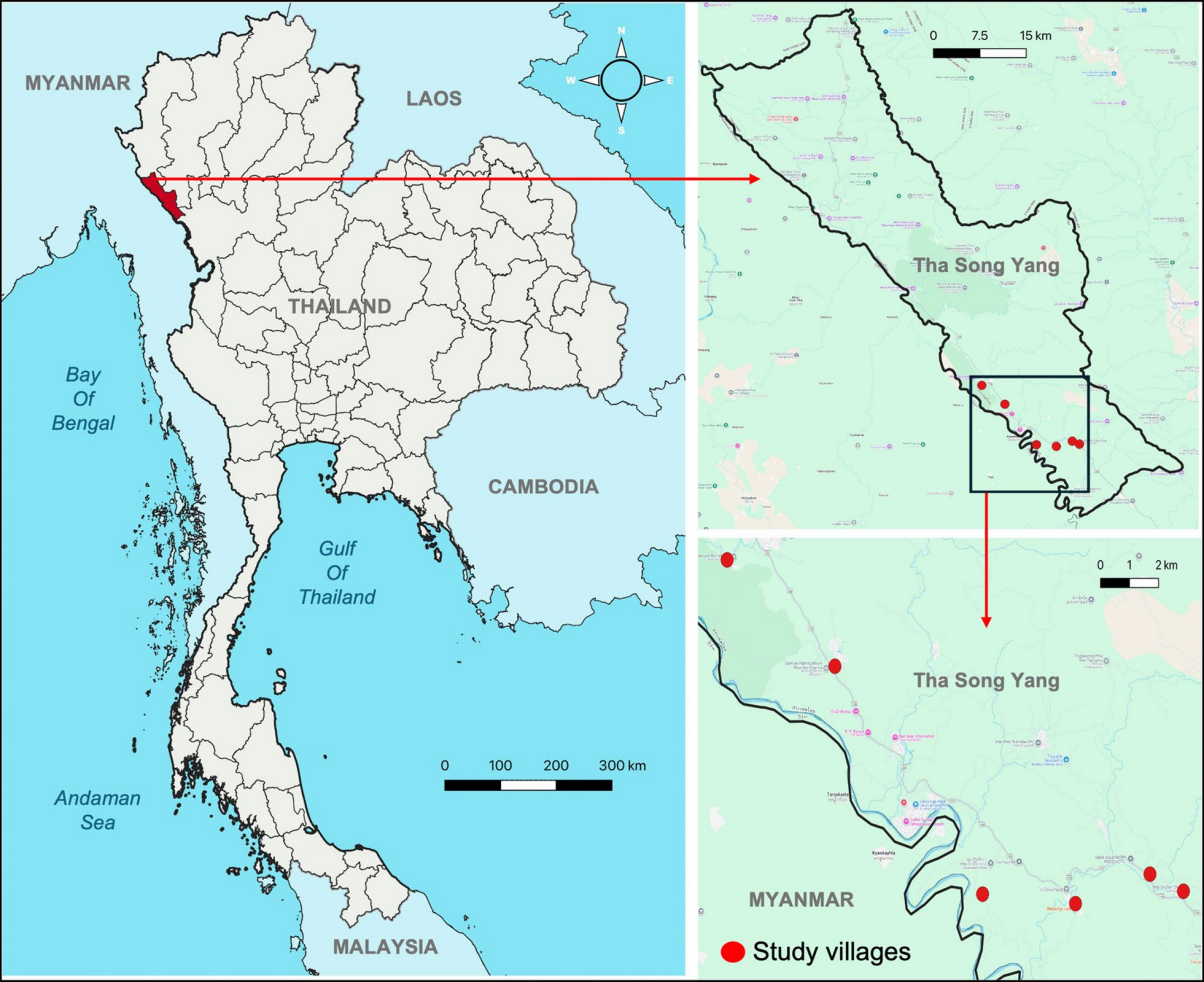
Location of the study district and six villages in northwestern Thailand. The map was created using QGIS (version 3.34.2-Prizren). Administrative boundaries and geographic features were adapted from publicly available shapefiles (https://gadm.org/)

Thailand comprises 76 provinces, with malaria transmission primarily concentrated along international borders, especially in the northwestern region. Tak Province, which borders southeastern Myanmar, was purposively selected due to its consistently high malaria incidence and recent resurgence. In 2024, Tak reported approximately 7300 malaria cases, accounting for 48% of all reported cases in the country [2]. Among the nine districts in Tak, Tha Song Yang, an area with both high malaria incidence in 2024 and a large population of Myanmar migrants, was selected as the study site (Fig. 1). Of the 56 villages in this district, six with the highest numbers of reported malaria cases and substantial migrant presence were chosen in consultation with provincial and district malaria control officers.

### Study participants

The study population consisted of short-term Myanmar migrants who self-reported arrival in Thailand within the previous six months. Prior to enrollment, local healthcare providers and village health volunteers (VHVs) compiled a list of eligible migrants within their catchment areas. Although the lists were compiled through community outreach and local knowledge rather than formal registries, VHVs in the study area routinely engage with migrant households as part of malaria prevention activities. This approach was therefore considered an effective method to identify eligible migrants within the selected villages. VHVs, who routinely assist with malaria-related outreach in their assigned local area, typically covering 10–15 households, played a central role in identifying potential participants. From these lists, systematic sampling was applied to recruit participants. The sampling interval was calculated by dividing the total number of eligible migrants identified in each village by the target sample size for that village (approximately 50 participants), and every 3rd individual on the list was approached for recruitment.

The sample size was calculated using the single-population proportion formula [33], assuming a 20% seroprevalence among migrants at risk of malaria, based on previous serological studies in mobile populations in the GMS [30, 31, 34] and used as a conservative estimate in the absence of specific data for short-term Myanmar migrants in Thailand, with a 5% margin of error, and a 10% non-response rate. This yielded a required sample size of 300 short-term migrants. Accordingly, approximately 50 participants were recruited from each of the six selected villages. Eligible participants were Myanmar nationals aged 18 years or older who had resided in Thailand for less than six months, regardless of gender or migration status. Individuals who self-reported a malaria diagnosis in the past month were excluded to avoid potential overestimation of antibody prevalence.

### Data and blood sample collection

Two types of data were collected in this study: questionnaire-based sociodemographic and behavioral information, and blood samples for serological testing.

*Questionnaire data*. Sociodemographic and behavioral data were collected using a structured questionnaire adapted from previous studies [20, 35–39]. The original questionnaire was developed in English as part of the study protocol and then translated into Thai for use by field research staff. Although participants were Myanmar migrants, the majority in the study area belong to the Kayin ethnic group and primarily speak Karen languages, which include multiple dialects and have limited standardized written forms. In addition, many participants have limited formal literacy. Therefore, the questionnaire was administered orally rather than as a written self-completed survey. Trained VHVs, who were fluent in both Thai and Karen and familiar with local dialects, conducted the interviews and translated the questions verbally into Karen during household visits to ensure participant comprehension.

The questionnaire collected information on gender, age, education level, ethnicity, occupation, workplace stability (categorized as permanent, seasonal, or temporary), estimated monthly income, number of days since arrival in Thailand, frequency of return to Myanmar, and living arrangements (alone, with family, or with friends/colleagues). Participants were also asked about their lifetime history of malaria and the estimated travel time to the nearest health facility. As previously reported, the questionnaire also included sections assessing malaria-related knowledge (transmission, prevention, and treatment), attitudes toward early diagnosis and treatment, and care-seeking behaviors. The full questionnaire has been published as a supplementary file in a previous study [40].

*Blood sample collection*. Dried blood spots (DBSs) were obtained from each participant using finger-prick blood samples collected onto pre-labeled Whatman filter papers. Two DBS cards were collected per participant. Samples were air-dried, individually sealed in envelopes, and transported to the Mahidol Vivax Research Unit (MVRU) in Bangkok for laboratory analysis.

*Data collection process*. To facilitate community-based data and sample collection, ten experienced VHVs were hired as field research assistants. All VHVs were fluent in both Thai and Karen and had previous experience with health-related outreach activities. They completed a one-day training session that covered study objectives, informed consent procedures, proper administration of the questionnaire, DBS collection, and principles of good clinical practice. The training included practical role-play exercises, during which the research team confirmed that the translation and delivery of questions were consistent with the intended meaning. Trained VHVs conducted structured household visits to the residences of eligible migrants. Each survey session lasted approximately 30 min and included both questionnaire administration and blood sample collection.

### Serological assays

Dried blood spots (DBSs) were stored in sealed envelopes at room temperature under continuous air conditioning (approximately 25 °C) for up to three months before laboratory processing. A previous study has demonstrated that IgG antibodies remain stable in DBSs under similar storage conditions [41]. All samples were initially screened by nested polymerase chain reaction (PCR) for identification of the *Plasmodium* genus and for *P. vivax*-specific infection. Six samples (three males and three females) tested positive for *P. vivax* DNA [40] and were excluded from the serological analysis to avoid bias from active infection, leaving 294 samples included in the present study.

Antibody responses to eight *P. vivax* antigens were measured using a multiplexed bead-based assay on the Luminex platform, following established protocols [42]. The selected antigens represent different parasite stages and functional roles and were designed to characterize recent exposure to *P. vivax* parasites [29]. Here, recent exposure was defined as occurring within the past nine months. Since IgG antibodies typically take several weeks to become detectable following infection, exposure likely occurred between one and nine months prior, roughly corresponding to the previous rainy season, which is the primary malaria transmission period in this setting. These included erythrocyte binding protein (EBP), *P. vivax* fam-a protein (Pv-fam-a), merozoite surface proteins 1–19 (MSP1-19), 5 (MSP5), and 8 (MSP8), protein export translocon 150 (PTEX150), cysteine-rich small secreted protein (CSS), and reticulocyte binding protein 2b (RBP2b) (Additional file 1: Table S1). These *P. vivax* proteins were coupled to magnetic microbeads at WEHI as previously described [42], then shipped to MVRU.

DBS samples were eluted in 150 µL of 0.5% v/v NP-40 1X PBS, 1X protease inhibitor cocktail (Sigma-Aldrich) and 0.1% w/v sodium azide (Sigma-Aldrich), overnight at room temperature, and considered equivalent to a 1:40 dilution of plasma. The eluants were then further diluted 2.5 times, yielding an approximate final plasma dilution of 1:100. Samples and coupled microbeads were incubated together for 30 min, followed by illumination of bound antibody using an anti-human IgG antibody conjugated to PE (Jackson ImmunoResearch 709116098). Assay plates were read on a BioPlex-200 instrument. Median Fluorescence Intensity (MFI) values were recorded for each antigen and converted to Relative Antibody Units (RAUs) using a standard curve generated from a serial dilution of positive control sera derived from Ethiopian individuals with confirmed *P. vivax* exposure, using the online R Shiny App [43]. Negative control samples from malaria-naïve individuals were also included to monitor background reactivity and assay performance. This standardization enabled comparability across plates and assays.

Binary classification of predicted recent *P. vivax* exposure within the prior nine months was performed using the previously developed random forest classification algorithm implemented in the same R Shiny App. In this approach, continuous log_10_-transformed RAU values for the eight *P. vivax* antigens are used simultaneously as predictors in the model rather than applying antigen-specific seropositivity cut-offs. The algorithm integrates responses across all antigens to estimate the probability of recent exposure based on patterns learned from previously validated training datasets. Individuals were classified as predicted recently exposed or not exposed according to the selected specificity threshold.

### Data analysis

RAUs were log_10_-transformed before analysis. Distributions of antibody levels were visualized using violin plots overlaid with boxplots showing medians and interquartile ranges. Additional boxplots were generated to compare antibody responses across key demographic and behavioral subgroups. MSP8 was excluded from these descriptive subgroup comparisons because its values were clustered near the lower detection limit with minimal variability across participants, which limited its usefulness for visualization. However, MSP8 was retained in the random forest classification and composite antibody score calculations because the validated algorithm incorporates responses to all eight antigens. Participant characteristics were summarized using frequencies and percentages for categorical variables and means with standard deviations and ranges for continuous variables.

Individuals were classified as seropositive or seronegative for *P. vivax* exposure using a previously validated RF classification algorithm. This model, designed for low-transmission settings, predicts recent *P. vivax* exposure within a nine-month timeframe. While the migration history of our study population is uncertain and may involve higher cumulative malaria exposure than typical in low-transmission contexts, we applied this method to provide a standardized measure of likely recent exposure. Among the three available specificity thresholds (81%, 90%, and 95%) for the classification algorithm, the 95% specificity cutoff was applied in this study. This threshold was chosen because the study population comprises short-term Myanmar migrants who had been in Thailand for less than six months and originated from areas with historically high malaria endemicity, where maximizing specificity helps reduce false positives.

Crude and adjusted odds ratios (cORs, aORs) with 95% confidence intervals (CIs) were estimated using logistic regression to identify factors associated with predicted recent *P. vivax* exposure. All covariates were included in multivariable models irrespective of their significance in univariable models, based on theoretical relevance and prior literature. Multicollinearity was assessed using variance inflation factors (VIF), with no evidence of strong collinearity (all adjusted GVIF^(1/2df) < 1.6). Model fit was acceptable (Hosmer–Lemeshow χ^2^ = 5.67, df = 8, *p* = 0.684), and discriminatory ability was moderate (AUC = 0.754).

To complement the binary classification approach, we also analyzed antibody responses as a continuous outcome. All eight antigens were retained in the composite score calculation to maintain consistency with the antigen panel used in the validated random forest classification algorithm. Because serological markers differ in measurement scale, log_10_-transformed RAUs were standardized (z-scores) and summed across all eight markers to create a composite antibody response score, representing overall seroreactivity. This approach aimed to capture cumulative exposure and to account for the possibility that the migrant population may have experienced higher levels of prior malaria exposure than assumed in the RF model. Multivariable linear regression was performed to assess associations between the composite antibody response score and individual-level sociodemographic and behavioral factors. The model assumed a Gaussian distribution. Multicollinearity diagnostics indicated no concerns (all adjusted GVIF < 2). Residual analyses revealed non-normality (Shapiro–Wilk test *p* < 0.001) and some indication of heteroscedasticity (Breusch–Pagan BP = 30.98, df = 22, *p* = 0.097); therefore, robust standard errors were applied. Sensitivity analyses excluding high-leverage observations (Cook’s distance > 4/n) yielded similar results, supporting the robustness of the findings. The final model explained approximately 19.4% of the variance in antibody responses (adjusted R^2^ = 0.194).

Two complementary outcome measures were analyzed. The binary outcome derived from the random forest algorithm was used to identify determinants of predicted recent *P. vivax* exposure within the preceding nine months. In contrast, the composite antibody score represents overall seroreactivity across multiple antigens and may reflect cumulative exposure over longer periods. These complementary approaches allowed exploration of both recent and longer-term exposure patterns. All analyses were performed using R version 4.5.1 (2025-06-13) in RStudio version 2025.05.1 + 513.

## Results

### Participant profile and migration characteristics

The study enrolled 294 short-term Myanmar migrants, with a balanced gender distribution (52.7% female) and a mean age of 35 years. The majority were ethnic Karen people (71.1%) and with no formal education (71.1%), reflecting a potentially socially vulnerable population. Most participants had unstable employment: 59.2% reported temporary work, and only 8.2% were in permanent positions. Daily wage labor was the most common occupation (38.4%), followed by other informal jobs such as construction and hotel work (34.7%). The mean monthly income was 4,000 THB (approximately 120 USD), highlighting substantial economic hardship. Participants reported a mean duration of stay in Thailand of 89 days (range: 3–168), and nearly half reported frequent cross-border movement (25.2% returned to Myanmar weekly and 17.7% monthly). Living arrangements were mixed: 39.5% lived alone and 39.8% lived with family. A prior history of malaria was reported by 39.5% of participants.

Although the mean travel time to a health facility was relatively short (19 min), the mean scores for malaria knowledge (11.5 ± 2.6 out of 18), attitudes (32.8 ± 3.7 out of 44), and care-seeking behavior (10.9 ± 2.4 out of 16) suggest room for improvement in awareness and timely health engagement (Table 1). These continuous scores were subsequently used to classify participants into distinct KAP subgroups, using the mean score as a cutoff point to identify individuals with high versus low knowledge, positive versus negative attitudes, and appropriate versus inappropriate care-seeking behavior.

**Table 1:** Sociodemographic characteristics of participants (n = 294)

Characteristic	n (%) or Mean ± SD (Range)
Gender	
Female	155 (52.7)
Male	139 (47.3)
Age (years)	35 ± 15 (18–78)
Education level	
No formal education	209 (71.1)
Primary school	57 (19.4)
Secondary school and above	28 (9.5)
Ethnicity	
Burmese	85 (28.9)
Karen	209 (71.1)
Occupation	
Agricultural	64 (21.8)
Daily wage	113 (38.4)
Unemployed	15 (5.1)
Other (construction, waiter/waitress, hotel)	102 (34.7)
Workplace stability	
Permanent	24 (8.2)
Seasonal	96 (32.7)
Temporary	174 (59.2)
Monthly income (THB)*	4,000 ± 2,000 (500–20,000)
Days in Thailand	89 ± 49 (3–168)
Return frequency to Myanmar	
Monthly	52 (17.7)
Once every few months	38 (12.9)
Weekly	74 (25.2)
Rarely	130 (44.2)
Living arrangement	
Alone	116 (39.5)
With family	117 (39.8)
With friends/colleagues	61 (20.7)
Malaria history	
Yes	116 (39.5)
No	178 (60.5)
Distance to health facility (minutes)	19 ± 11 (5–60)
Knowledge score	11.5 ± 2.6 (4–18)
Attitude score	32.8 ± 3.7 (27–44)
Care-seeking score	10.9 ± 2.4 (4–16)

^*^33 THB ≈ 1 USD

### Distribution of antibody responses to *P. vivax* antigens

The distributions of log_10_-transformed relative antibody levels varied by antigen (Additional file 1: Fig. S1). Median responses were − 3.32 (IQR: − 3.48 to − 3.09) for RBP2b, − 3.53 (− 3.65 to − 3.35) for CSS, and − 3.66 (− 3.82 to − 3.46) for Pv-fam-a. EBP and PTEX150 both had a median of − 3.98, with interquartile ranges of − 4.43 to − 3.44 and − 4.09 to − 3.81, respectively. MSP1-19 showed a median of − 4.14 (− 4.45 to − 3.61). In contrast, both MSP5 and MSP8 displayed median values close to the lower detection limit [− 4.65 (− 4.69 to − 4.03) and − 4.69 (− 4.69 to − 4.69), respectively], indicating minimal detectable responses for these markers. Although MSP5 and MSP8 both showed relatively low responses, MSP8 displayed minimal variability across participants and was therefore excluded from subgroup visualization to improve interpretability of the plots. While absolute magnitudes cannot be directly compared across antigens due to the assay setup (i.e. differing protein amounts coupled to beads), they are partially standardized by using a common positive control pool. The observed distributions showed antigen-specific patterns in the breadth and variability of antibody reactivity within this migrant population.

### Factors associated with recent *P. vivax* exposure

Among the 294 participants, 104 (35.4%) were classified as having predicted recent *P. vivax* exposure using the previously validated RF classification algorithm. This classification was based on the combined pattern of continuous antibody responses across the eight *P. vivax* antigens rather than antigen-specific seropositivity cut-offs (Table 2).

**Table 2:** Logistic regression models for factors associated with recent *P. vivax* exposure among short-term Myanmar migrants (n = 294)

Characteristic	Sero (+) (n = 104)	Sero (−) (n = 190)	cOR (95% CI)	aOR (95% CI)
	n (%)	n (%)		
Gender				
Female	61 (39.4)	94 (60.6)	Ref	Ref
Male	43 (30.9)	96 (69.1)	0.69 (0.42, 1.12)	1.12 (0.61, 2.05)
Age (years)				
< 20	17 (34.7)	32 (65.3)	Ref	Ref
20–29	22 (25.0)	66 (75.0)	0.63 (0.29, 1.35)	0.93 (0.38, 2.28)
30–39	26 (44.8)	32 (55.2)	1.53 (0.70, 3.39)	3.11 (1.15, 8.80)
≥ 40	39 (39.4)	60 (60.6)	1.22 (0.60, 2.53)	1.80 (0.70, 4.80)
Education level				
No formal education	73 (34.9)	136 (65.1)	Ref	Ref
Primary school	16 (28.1)	41 (71.9)	0.73 (0.37, 1.36)	0.72 (0.31, 1.66)
Secondary school and above	15 (53.6)	13 (46.4)	2.15 (0.97, 4.83)	3.28 (1.09, 10.3)
Ethnicity				
Burmese	20 (23.5)	65 (76.5)	Ref	Ref
Karen	84 (40.2)	125 (59.8)	2.18 (1.25, 3.95)	3.68 (1.42, 10.0)
Occupation				
Unemployed	6 (40.0)	9 (60.0)	Ref	Ref
Agricultural	20 (31.2)	44 (68.8)	0.68 (0.22, 2.27)	1.34 (0.28, 6.66)
Daily wage	43 (38.1)	70 (61.9)	0.92 (0.31, 2.92)	1.11 (0.26, 5.00)
Other (construction, waiter/waitress, hotel)	35 (34.3)	67 (65.7)	0.78 (0.26, 2.50)	0.98 (0.21, 4.84)
Workplace stability				
Permanent	13 (54.2)	11 (45.8)	Ref	Ref
Seasonal	31 (32.3)	65 (67.7)	0.40 (0.16, 1.00)	0.28 (0.08, 0.95)
Temporary	60 (34.5)	115 (65.5)	0.45 (0.18, 1.05)	0.26 (0.08, 0.84)
Monthly income (THB)				
≤ 3,000	59 (44.7)	73 (55.3)	Ref	Ref
3001–5000	34 (29.6)	81 (70.4)	0.52 (0.30, 0.88)	0.37 (0.19, 0.71)
≥ 5000	11 (23.4)	36 (76.6)	0.38 (0.17, 0.79)	0.17 (0.06, 0.45)
Days in Thailand				
< 60	26 (32.1)	55 (67.9)	Ref	Ref
60–89	16 (32.7)	33 (67.3)	1.03 (0.48, 2.18)	0.82 (0.30, 2.21)
90–179	62 (37.8)	102 (62.2)	1.29 (0.74, 2.28)	0.95 (0.36, 2.51)
Return frequency to Myanmar				
Rarely	51 (39.2)	79 (60.8)	Ref	Ref
Monthly	17 (32.7)	35 (67.3)	0.75 (0.38, 1.47)	0.82 (0.35, 1.86)
Once every few months	18 (47.4)	20 (52.6)	1.39 (0.67, 2.89)	1.36 (0.58, 3.23)
Weekly	18 (24.3)	56 (75.7)	0.50 (0.26, 0.93)	0.60 (0.23, 1.54)
Living arrangement				
Alone	36 (31.0)	80 (69.0)	Ref	Ref
With family	48 (41.0)	69 (59.0)	1.55 (0.90, 2.66)	1.68 (0.85, 3.36)
With friends/colleagues	20 (32.8)	41 (67.2)	1.08 (0.55, 2.09)	1.26 (0.55, 2.88)
Malaria history				
No	62 (34.8)	116 (65.2)	Ref	Ref
Yes	42 (36.2)	74 (63.8)	1.06 (0.65, 1.73)	0.91 (0.50, 1.67)
Distance to health facility (minutes)				
≤ 15	29 (33.7)	57 (66.3)	Ref	Ref
15–29	55 (41.0)	79 (59.0)	1.37 (0.78, 2.42)	2.07 (1.00, 4.43)
30–59	20 (27.8)	52 (72.2)	0.76 (0.38, 1.49)	1.87 (0.66, 5.50)
≥ 60	0 (0)	2 (100)	-	-
Knowledge score				
High	56 (37.3)	94 (62.7)	Ref	Ref
Low	48 (33.3)	96 (66.7)	0.84 (0.52, 1.35)	1.01 (0.53, 1.92)
Attitude score				
Positive	62 (36.3)	109 (63.7)	Ref	Ref
Negative	42 (34.1)	81 (65.9)	0.91 (0.56, 1.48)	2.02 (1.04, 4.03)
Care-seeking score				
Appropriate	70 (38.7)	111 (61.3)	Ref	Ref
Inappropriate	34 (30.1)	79 (69.9)	0.68 (0.41, 1.12)	0.66 (0.34, 1.25)

33 THB ≈ 1 USD; cOR: Crude odds ratio; aOR: Adjusted odds ratio; 95% CI 95% confidence interval; Ref.: Reference; Odds ratios were not estimated for the ≥ 60-min category due to the absence of seropositive cases

Logistic regression analysis identified several demographic and behavioral factors significantly associated with recent *P. vivax* exposure among short-term Myanmar migrants. In the adjusted model, participants aged 30–39 years had over three times the odds of predicted recent *P. vivax* exposure compared with those younger than 20 years (aOR = 3.11, 95% CI 1.15–8.80). Secondary education or higher was also associated with greater odds of predicted recent *P. vivax* exposure than no formal education (aOR = 3.28, 95% CI 1.09–10.3). Karen ethnicity was strongly associated with higher odds of predicted recent *P. vivax* exposure than Burmese (aOR = 3.68, 95% CI 1.42–10.0).

Indicators of socioeconomic position and work stability were inversely related to predicted recent *P. vivax* exposure. Both seasonal (aOR = 0.28, 95% CI 0.08–0.95) and temporary work (aOR = 0.26, 95% CI 0.08–0.84) were associated with significantly lower odds of predicted recent *P. vivax* exposure compared to permanent employment. Similarly, higher monthly income was associated with lower odds of predicted recent exposure; migrants earning > 5,000 THB had markedly reduced odds of predicted recent *P. vivax* exposure compared to those earning ≤ 3,000 THB (aOR = 0.17, 95% CI 0.06–0.45). Additional associations were observed with behavioral and health-related factors. Living more than 15 min from a health facility was associated with increased odds of predicted recent *P. vivax* exposure (aOR = 2.07, 95% CI 1.00–4.43). Moreover, negative attitudes toward malaria prevention and care were associated with higher odds of predicted recent *P. vivax* exposure (aOR = 2.02, 95% CI 1.04–4.03). No significant associations were found for gender, malaria history, frequency of return to Myanmar, or malaria knowledge and care-seeking scores.

### Factors associated with antibody responses and composite antibody scores

**Fig. 2:**
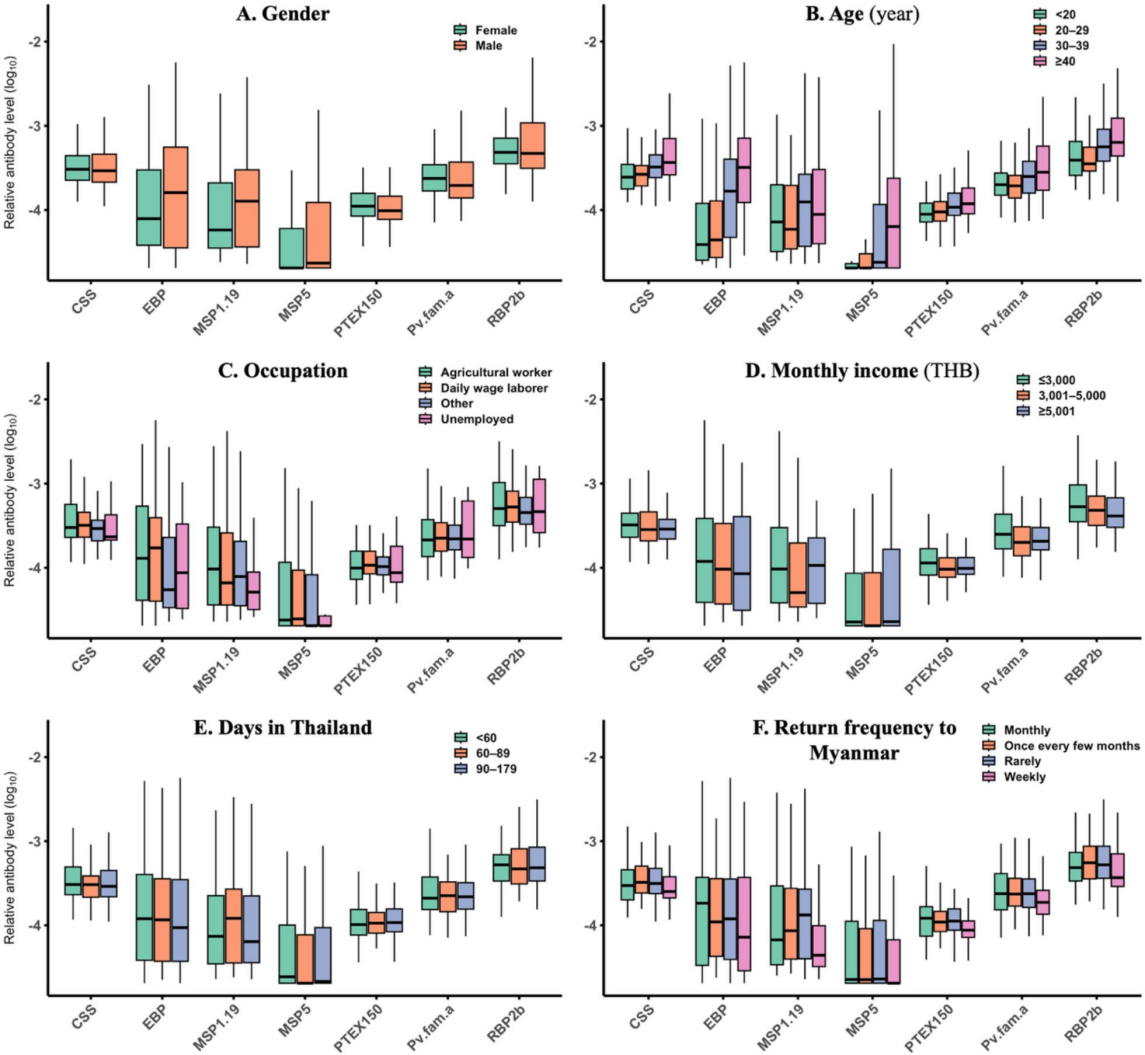
Relative antibody levels (log_10_-transformed RAU) against *P. vivax* antigens across major demographic and behavioral subgroups. Each panel displays boxplots stratified by a specific covariate: **A** gender, **B** age group, **C** occupation, **D** monthly income, **E** duration of stay in Thailand, and **F** frequency of return to Myanmar. Boxplots show the median (horizontal line), interquartile range (box), and whiskers extending to 1.5 times the interquartile range. Antibody responses are displayed separately for each *P. vivax* antigen

Descriptive comparisons of log_10_-transformed antibody responses across demographic and behavioral subgroups revealed several patterns (Fig. 2). Antibody distributions differed across age groups, gender, and occupational categories, with higher responses generally observed among older individuals and males. Patterns related to agricultural work, income level, duration of stay in Thailand, and cross-border mobility were less clearly differentiated in the visual distributions. These plots are intended to illustrate overall antibody distribution patterns, while formal statistical associations were evaluated using regression models (Tables 2 and 3). Other factors, including distance to the nearest health facility, malaria-related knowledge, attitudes, and care-seeking behaviors, education level, ethnicity, workplace stability, living arrangement, and malaria history, showed broadly comparable distributions and are presented in Additional file 1: Fig. S2.

**Table 3:** Multivariable linear regression of factors associated with composite scores of standardized log_10_-transformed antibody levels to *P. vivax* antigens among short-term Myanmar migrants (n = 294)

Characteristic	Estimates (β)	95% CI	*p*-value
Gender			
Female	Ref		
Male	1.17	0.07, 2.28	0.038*
Age (per year)	0.12	0.08, 0.17	< 0.001*
Education level			
No formal education	Ref		
Primary school	− 1.21	− 2.70, 0.28	0.11
Secondary school and above	− 1.35	− 3.14, 0.45	0.14
Ethnicity			
Burmese	Ref		
Karen	1.93	− 0.30, 4.16	0.089
Occupation			
Unemployed	Ref		
Agricultural	2.58	0.01, 5.14	0.049*
Daily wage	1.69	− 0.83, 4.20	0.20
Other (construction, waiter/waitress, hotel)	2.43	− 0.17, 5.03	0.067
Workplace stability			
Permanent	Ref		
Seasonal	− 1.24	− 3.37, 0.89	0.30
Temporary	− 1.75	− 3.54, 0.04	0.055
Monthly income (per 10,000THB)	− 3.83	− 6.28, − 1.37	0.002*
Days in Thailand	− 0.02	− 0.04, − 0.00	0.025*
Return frequency to Myanmar			
Rarely	Ref		
Monthly	0.42	− 1.28, 2.21	0.60
Once every few months	0.62	− 1.16, 2.40	0.50
Weekly	− 2.44	− 4.14, − 0.74	0.005*
Living arrangement			
Alone	Ref		
With family	1.27	− 0.06, 2.60	0.061
With friends/colleagues	0.57	− 0.78, 1.93	0.40
Malaria history			
No	Ref		
Yes	− 0.65	− 1.85, 0.56	0.30
Distance to health facility (per minute)	0.02	− 0.06, 0.09	0.70
Knowledge score	− 0.05	− 0.29, 0.19	0.70
Attitude score	0.05	− 0.18, 0.28	0.70
Care-seeking score	0.16	− 0.16, 0.47	0.30

^*^Statistical significance at *p* < 0.05; 33 THB ≈ 1 USD; 95% CI 95% confidence interval; Ref.: Reference

Multivariable linear regression analysis was conducted using a continuous outcome, defined as a composite antibody score calculated by summing standardized log_10_-transformed responses across all eight *P. vivax* antigens. GLMs with a gaussian distribution and robust standard errors were applied to identify factors significantly associated with antibody levels. Males had significantly higher composite scores compared to females (β = 1.17, 95% CI 0.07 to 2.28, *p* = 0.038). Increasing age was also associated with an elevated composite score, with each additional year corresponding to a 0.12 standard deviation increase (95% CI 0.08 to 0.17, *p* < 0.001). Participants working in agriculture had significantly higher composite scores than those who were unemployed (β = 2.58, 95% CI 0.01 to 5.14, *p* = 0.049). Conversely, higher monthly income was associated with lower composite scores (β = − 3.83 per 10,000 THB, 95% CI –6.28 to –1.37, *p* = 0.002), as was longer duration of stay in Thailand (β = − 0.02 per day, 95% CI –0.04 to –0.00, *p* = 0.025). Frequent cross-border mobility was also a significant factor; participants who returned to Myanmar weekly had lower composite scores compared to those who rarely returned (β = − 2.44, 95% CI –4.14 to –0.74, *p* = 0.005). No significant associations were observed for education level, ethnicity, workplace stability, living arrangement, malaria history, or malaria-related knowledge, attitudes, and care-seeking scores (Table 3).

### Discussion

Thailand and Myanmar share approximately 550 km of border across nine provinces in the Thailand’s northwestern region and Myanmar’s southeastern region. Both sides have long demonstrated malaria receptivity due to ecological suitability for major *Anopheles* vectors [2, 44]. Migrant populations in these border regions remain at heightened risk of *P. vivax* infection. The risk arises not only from limited and irregular access to malaria services, including early diagnosis, preventive measures, and health information, but also from the potential for individuals to harbor low-density asymptomatic parasitemia and dormant liver-stage parasites that are difficult to detect through routine surveillance [13, 40]. Both conditions contribute to onward transmission, and therefore serological surveillance is highly valuable among such populations to highlight risk of exposure, risk-factors for exposure, and to potentially guide targeted treatment.

This study measured antibody responses to eight *P. vivax*-specific markers and applied two analytic approaches: a validated recent-exposure classification algorithm [29] and a composite measurement of balanced antibody quantities. Using both approaches allowed a more comprehensive representation of recent and cumulative exposure patterns in a highly mobile population. Even at the highest available specificity threshold of 95%, the proportion classified as seropositive (35.4%) was higher than that previously reported among mobile populations in Cambodia (31.0%) [34] and Thai nationals residing in endemic areas of Thailand (15.6%) [30]. This is likely influenced by the nature of the study population: short-term Myanmar migrants originating from regions where malaria transmission has increased since 2022 due to political instability and disruptions to health services [16]. These findings suggest substantial recent exposure and are consistent with prior infection that may place some migrants at risk of hypnozoite carriage, which could contribute to a high probability of relapse and continued vulnerability to infection, as well as supporting onward transmission.

Several predictors were associated with predicted recent *P. vivax* exposure in the algorithm-based analysis. Individuals who have lived in malaria-endemic settings may acquire partial immunity from repeated infections, and therefore older adults generally demonstrate higher antibody levels than younger individuals due to cumulative lifetime infections [30, 45, 46]. Occupational exposure further increases risk among adults working in environments where bed net use is impractical, including agricultural fields, plantations, or forest-adjacent areas [47]. These patterns were evident in the current study, with adults aged 30 to 39 exhibiting higher predicted recent *P. vivax* exposure, and composite antibody levels showing an overall increase with age. While the serological algorithm is optimized to detect exposures within the past nine months, it remains possible that individuals with long-standing, repeated exposures, such as older adults, may retain elevated antibody levels that marginally influence algorithm classification, even in the absence of very recent infection. These findings highlight the importance of targeted prevention strategies for working-age adults whose occupational environments may limit the consistent use of malaria protection.

Educational attainment did not exhibit a protective association; risk of predicted recent *P. vivax* exposure was higher in individuals with secondary school education compared to no education. Health literacy does not necessarily correspond to formal education [48]. For example, individuals with limited literacy may still receive malaria-related information through community networks, local health talks, or informal communication channels. Conversely, individuals with higher education may still be exposed due to occupational patterns or mosquito biting behavior before sleeping hours [8]. Language barriers among migrants in Thailand can further reduce access to health information [13]. Individuals with negative attitudes toward malaria demonstrated higher predicted recent *P. vivax* exposure. Previous experiences of non-severe *P. vivax* episodes, perceptions of low disease severity, or familiarity with effective treatment may lead to reduced engagement in preventive behaviors [13]. Daily economic priorities among migrant workers may also limit the time and attention available for malaria prevention. Moreover, the frequent occurrence of malaria in some communities may contribute to the normalization of its symptoms, whereby the disease is perceived as a routine aspect of life, diminishing motivation to adopt protective behaviors [49]. Such perceptions can undermine malaria control efforts, particularly where preventive measures require consistent personal adherence rather than one-time intervention. These observations indicate the need for malaria education strategies that are linguistically and contextually adapted, independent of educational background.

Ethnicity also demonstrated associations with predicted recent *P. vivax* exposure. Karen individuals, who historically reside in forested, geographically isolated areas along the border, showed higher predicted recent *P. vivax* exposure than Burmese individuals who generally originate from central Myanmar. Border regions inhabited by Karen populations have long-standing malaria transmission and instability [44]. However, increasing migration from central Myanmar in recent years suggests that exposure patterns may shift, and therefore malaria services should ensure equitable access across all migrant subgroups. Access to health services remains a critical determinant of malaria risk. Interestingly, predicted recent *P. vivax* exposure was higher among individuals living 15–29 min from the nearest health facilities compared to those < 15 min, while no difference was observed among those living 30–59 min away, potentially highlighting the existing of other confounding factors not accounted for. Individuals residing closer to health facilities may have more opportunities to receive free malaria diagnosis and treatment regardless of citizenship or documentation status, as well as access to preventive commodities such as long-lasting insecticidal nets, indoor residual spraying (IRS), and repellents [50, 51]. Short-term migrants are frequently excluded from local census systems and may therefore be missed during commodity distribution [16]. More flexible, community-based listing or rolling registration systems could help ensure that these groups are adequately reached.

Short-term migrants engaged in permanent or labor-intensive occupations exhibited higher predicted recent *P. vivax* exposure than seasonal migrants. However, because participants were enrolled within six months of arrival and the algorithm measures exposure over nine months, part of this exposure likely resulted from infections acquired before migration. Lower income was also associated with increased risk of predicted recent *P. vivax* exposure. Individuals with limited financial resources may reside in housing structures that provide inadequate protection against mosquitoes and are not feasible for IRS [52]. Socioeconomic vulnerability has consistently been associated with *P. vivax* transmission dynamics in the region [53].

The composite antibody measurements indicated higher antibody levels among males and agricultural workers, which aligns with cultural and economic labor patterns in which men disproportionately engage in occupations with higher mosquito exposure [25, 30]. Increasing age was also associated with higher composite antibody levels. This association likely reflects cumulative exposure to malaria over time rather than a direct age-related biological effect. Individuals who have lived longer in endemic environments may have experienced repeated infections, leading to progressively higher antibody responses. A longer duration of residence in Thailand and weekly returns to Myanmar were associated with lower antibody levels. Thailand continues to intensify border malaria control efforts as part of its elimination strategy, while Myanmar currently faces broad disruptions to its health infrastructure [1]. Therefore, individuals newly arriving from Myanmar may carry higher infection risk reflecting exposure prior to migration, while those who have resided in Thailand longer may gradually experience declining exposure under Thailand’s intensified malaria control efforts [25, 54]. Weekly returnees may also vary in time spent and in the use of preventive measures at their places of origin, which requires further investigation. It is important to acknowledge that the composite antibody measurement likely reflects longer-term exposure, while the algorithm-based classification reflects predicted recent *P. vivax* exposure. This difference likely explains the variation in predictors identified across the two models. The random forest algorithm used in this study was originally developed and validated in low-transmission settings, whereas migrants included in the present study may have experienced higher cumulative exposure in their places of origin in Myanmar prior to migration. In such contexts, long-lived antibody responses from repeated infections could theoretically increase the probability of misclassification if past exposure is interpreted as recent infection. To reduce this risk, we applied the most conservative classification threshold available (95% specificity), which prioritizes minimizing false-positive classifications. These differences could be further explored by incorporating additional markers of long-term exposure, such as *P. vivax* apical membrane antigen 1 (PvAMA1) [55].

The findings of this study have several implications for malaria elimination strategies in border regions. First, the observed associations with age, occupation, income, and mobility patterns suggest that surveillance and prevention efforts may benefit from targeting working-age migrants engaged in agricultural or other outdoor occupations. Second, the higher predicted recent exposure among individuals living farther from health facilities and those with negative malaria attitudes highlights the importance of strengthening community-based outreach and culturally appropriate health communication among migrant populations. Third, the substantial proportion of migrants classified as having predicted recent exposure despite negative PCR results underscores the potential value of serological surveillance as a complementary tool to routine case detection, particularly for identifying populations with recent or asymptomatic infections. Together, these findings support the need for migrant-inclusive malaria services and flexible surveillance strategies that account for cross-border mobility in elimination settings.

This study represents, to the best of current knowledge, the first serological assessment of *P. vivax* exposure among Myanmar migrants in Thailand using dual analytic approaches and incorporates a broad set of socioeconomic and behavioral predictors. The use of serological methods allowed detection of recent malaria exposure over the preceding nine months, whereas PCR, microscopy, and RDTs primarily identify only active infections. Since antibody responses often persist beyond the parasitemic phase, serology enables detection of recent transmission events, even within the constraints of a cross-sectional study design. However, several limitations warrant consideration. All data were self-reported, introducing the potential for recall or social desirability bias. Migration documentation status was not collected due to ethical concerns, although undocumented migrants may experience additional structural barriers. Because participant lists were compiled through VHV community networks rather than formal registries, some migrants who were less connected to local health services may have been underrepresented. While sampling was conducted district-wide to enhance representativeness, generalizability to other migrant populations or border regions may be limited. Due to logistical constraints, only adults aged 18 or older were included. Given age-related variation in malaria exposure, inclusion of children may have provided further insight. The cross-sectional design limits causal interpretation, although the serological algorithm captures exposure over the previous nine months, encompassing both major seasonal transmission peaks (June–July and November–December) [2]. In addition, individuals who reported a malaria diagnosis within the previous month were excluded to avoid potential inflation of antibody responses associated with active infection. This approach may have resulted in a conservative estimate of antibody levels and predicted recent exposure in the study population.

## Conclusions

The findings indicate substantial recent and cumulative *P. vivax* exposure among short-term Myanmar migrants along the Thailand–Myanmar border, reflecting infections likely acquired prior to migration as well as ongoing vulnerability after arrival. Strengthening migrant-inclusive malaria services is essential, including proactive screening, culturally and linguistically tailored health communication, and equitable access to preventive commodities. Serological surveillance provides meaningful additional insight beyond routine case detection by identifying individuals and groups at increased risk who may harbor asymptomatic or relapsing infections. Broader integration of serological tools could support more targeted and effective malaria elimination strategies in border regions and contribute to continued progress toward Thailand’s national malaria elimination goal of 2030.

## Supplementary Information

Additional file 1.

## Data Availability

All data generated or analyzed during this study are included in the article. The de-identified raw dataset is available from the corresponding author upon reasonable request.
