# Supplementary material for: Sociodemographic and behavioral determinants of Plasmodium vivax-specific antibody responses among short-term Myanmar migrants in Thailand
Source: Malar J. 2026 Apr 29;25:237. doi: 10.1186/s12936-026-05921-0 (PMC13270664; doi:10.1186/s12936-026-05921-0)
Supplement: Supplementary file 1 — Additional file 1. [file 12936_2026_5921_MOESM1_ESM.docx]

**Supplementary Table 1** List of *P. vivax* antigens used as serological exposure markers, including amino acid regions of proteins expressed and protein expression system used.

| **Label** | **Name** | **PlasmoDB reference number** | **Amino acid region expressed** | **Expression system** |
| --- | --- | --- | --- | --- |
| CSS | Cysteine-rich, small, secreted protein | PVP01_1344100 | 22 – 381 | Mammalian Expi293F |
| EBP | Erythrocyte Binding Protein Region II | A0A0J9SL51* | 1 – 716 | Sf21 Insect cells |
| MSP1-19 | Merozoite Surface Protein 1 | PVX_099980 | 1623 – 1715 | *E. coli* |
| MSP5 | Merozoite Surface Protein 5 | PVX_003770 | 25 – 364 | *E. coli* |
| MSP8 | Merozoite Surface Protein 8 | PVX_097625 | 24 – 465 | Sf21 Insect cells |
| PTEX150 | *Plasmodium* Translocon of Exported Proteins component PTEX150 | PVX_084720 | 23 – 908 | Sf21 Insect cells |
| Pv-fam-a | Tryptophan-rich Antigen | PVX_096995 | 56 – 480 | *E. coli* |
| RBP2b | Reticulocyte Binding Protein 2b | PVX_094255 | 169 – 470 | *E. coli* |

*Uniprot reference number

**Supplementary Figure 1** Distribution of log₁₀-transformed relative antibody levels (RAU) for eight *P. vivax* serological markers. Each violin plot shows the distribution and central tendency of RAU. White boxes represent interquartile ranges with medians; violin outlines show the full distribution.

**
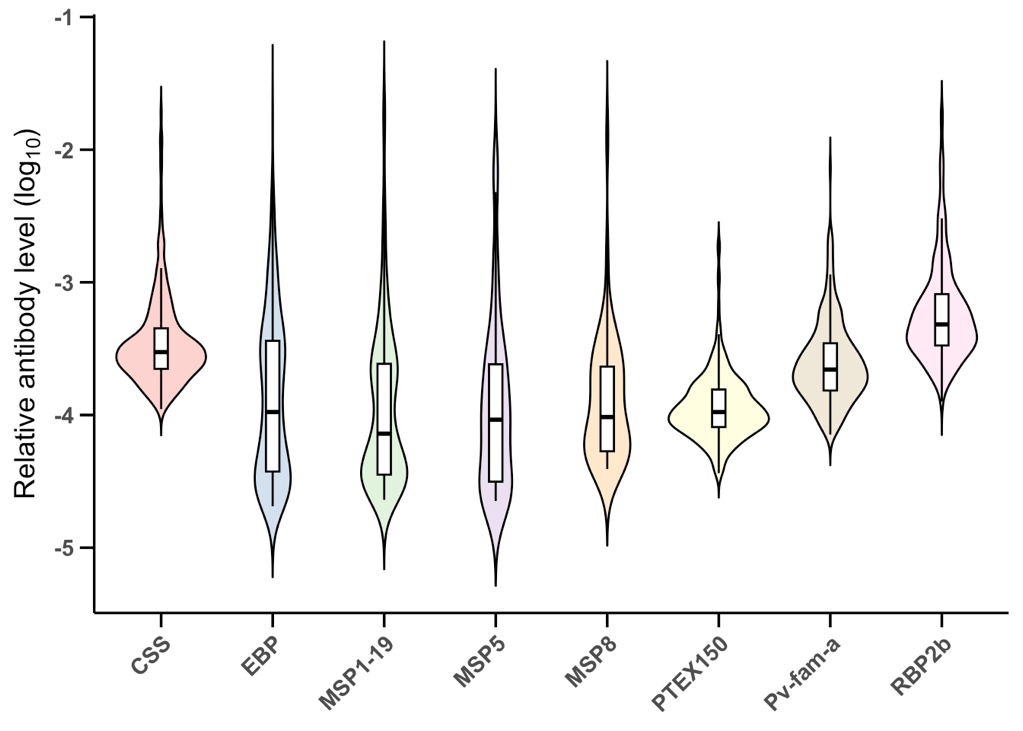
**

**Supplementary Figure 2** Relative antibody levels (log₁₀-transformed RAU) against *P. vivax* antigens across other demographic and behavioral subgroups. Each panel displays antibody responses stratified by a specific covariate. Boxplots represent the median, interquartile range (IQR), and whiskers extending to 1.5× the IQR.

**

**
